# Factors in relation with self–regulation of Hypertension, based on the Model of Goal Directed behavior in Yazd city

**Published:** 2011-02-25

**Authors:** M Baghianimoghadam, S Aivazi, SS Mzloomy, B Baghianimoghadam

**Affiliations:** * Associated professor, Faculty of Health, Shahid Sadoughi University of Medical sciences, YazdIran; ** Ms in Health Education, Shahid Sadoughi University of Medical Sciences, YazdIran; *** Associated professor, Faculty of Health, Shahid Sadoughi University of Medical sciences, YazdIran; **** Shahid Sadoughi University of Medical Sciences, YazdIran

**Keywords:** High blood pressure, Educational Model, Behavior, Regulation

## Abstract

**Introduction**: Hypertension is a chronic disease, which represents one of the most common public health problems in the world and afflicts about 28% of the North Americans, 44% of the Europeans and 26% of the East Mediterranean people aged 35 to 64 years. In Iran, about 11.5% of the people who are over 15 years old are afflicted. Hypertension is the most prevalent cardiovascular disease; it is the leading cause of stroke, heart attack, kidney disease and aortic aneurysm.

**Materials and Methods**: This is a cross–sectional study, carried out on 200 patients who were referred to the Health centers of Yazd city in center of Iran. We adapted tools from previous studies. All patients were asked to express their personal goals with respect to their hypertension values. The reliability and validity of the questionnaire were determined and their Alfa cronbach was (a=0.83).

**Results**: The mean number of years with hypertension was 8.7[+/–]7.6. Men and women showed moderately high means for attitudes, subjective norms, and positive anticipated emotions toward trying to reduce or maintain their blood pressure. The data indicated there is significantly different between the personnel and housewives, with p<0.001. In addition, the personnel's mean grade scores  for trying the self–regulation of their blood pressure was (15.9[+/–]4.08) and, in the housewives was (12.49[+/–]4.33).

**Conclusion**: The results of this study showed that the Model of Goal Behavior can explain more than 52% of the self–regulation of hypertension, so, this Model can be a basic Model for intervention in education, in order to decrease and control hypertension in patients.

## Introduction

Hypertension is a chronic cardiovascular disease, which is the leading cause of morbidities in human population, becoming a major public health problem [1]. About 28% of the North Americans, 44% of the Europeans and 26% of the East Mediterranean people, aged 35 to 64 years are involved [2]. Its prevalence is estimated to be of about 11.5% in the population over 15 years old, in Iran [3]. It is the leading cause of stroke, heart attack, kidney disease and aortic aneurysm [4]. 

Being a primary contributor to disability and mortality, hypertension cannot be cured but must be managed. Medication is the leading way to treating hypertension. Pressure control can substantially reduce the risk of stroke and heart attack. More than 100 different drugs are available for its treatment; all have proven efficacy, most have few side effects and many are formulated for once–daily dosing. Then, why do the reported rates of blood pressure control are so disappointing, and apparently not getting any better? Indeed, the success rate for the control of hypertension in the United States is only 27%, being even lower in England, France, and Germany [5].

Despite major financial and time investment, the treatment compliance of hypertension has remained low. The majority of hypertensive patients do not reach the current target Blood Pressure (BP) levels set by the international guidelines (self–monitoring).

One promising method to improve BP control in hypertensive patients is self–measurement of BP [6].Medical researchers report that the failure to regulate blood pressure originates in a poor compliance to medication regimens. However, blood pressure control is also related to the function of various health–compromising behaviors and health enhancing behaviors [7].

Clearly, the management of hypertension requires self-regulation, which is defined as the mental and physical process that a person manages in order to achieve a goal. Unfortunately, until now, studies have had limited inquiry to compliance with medication regimens and, have focused on correlations such as the health status. Little research exists about decision- making processes and social psychological aspect of the self-regulation of hypertension. Most of the discussions in the literature have been conceptual rather than empirical in nature [7]. The purpose of the present study is to investigate the decision–making process and the effort expanded in the self–management of hypertension and to provide guidance regarding the evaluation of decision–making models in the hypertension context. In order to thoroughly examine the hypertension management, there is a need to scrutinize goals, as well as actions, and to consider dynamic factors such as counterfactual thinking and feedbacks in the form of anticipated consequences of goal attainment and goal failure. As a result, we decided to apply the model of goal–directed behavior.

### Model of Goal–directed Behavior

To better explain goal striving, Bagozzi, Baumgartner, and Pieter [8] introduced anticipated emotions as predictors of volitions to act. They explained that people, when deliberating to act or not in a goal–directed situation, take into account the emotional consequences of both achieving and not achieving a sought after goal. Unlike passive attitude, subjective norms, and perceived behavioral control, the anticipated emotion functions dynamically in a self–regulatory sense, in response to the actual or imagined feedback [9,10]. That is, for a goal that one contemplates, one appraises the consequences of achieving and not achieving that goal, with corresponding positive and negative emotions arising. A comparison is made between one's goal as a standard or reference value and an estimate is made concerning how one would feel after achieving and failing to achieve the goal. The anticipated emotions finally function as to influence decision making by pressing for a decision that promotes positive emotions and avoids negative emotions

The processes behind the functioning of anticipated emotions are counterfactual thinking but might be termed as prefectural to stress the expected, forward–looking aspects of the thought processes. Individuals may think about imaginary alternatives to events in terms of the implications of these events for the future. People's behaviors may well be determined by what they imply for the future [11]. Thus, anticipated negative outcomes and experiences, as a function of imagined goal failure, are punishing and distressing, so, patients should be motivated to avoid them, by putting forth more effort to goal pursuit. Likewise, anticipated positive outcomes and experiences, as a function of imagined goal achievement, are rewarding and pleasant, so patients should be motivated to approach them by putting forth greater effort at goal striving. Evidence for the role of positive or negative anticipated emotions in decision–making can be found in Perugini and Conner [12].

The MGB also includes past behavior as a predictor of desires, intentions, and trying. This yields two benefits. First, past behavior serves as a control for unmeasured determinants of behavior that may have been stable over time in their effects. Second, past behavior may reflect habitual processes. A recent meta-analysis for the role of past behavior supports these claims, as do a number of recent studies [13]. 

**Graph 1:**
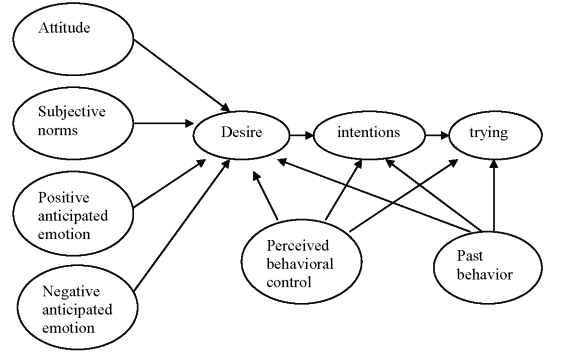
Model of Goal-Directed Behavior

## Materials|Methods

This was a cross–sectional study implemented on 200 patients who were referred to Health centers of Yazd city in center of Iran. We used some tools from the previous studies [14–17]. Patients had to meet the following criteria: 30 years of age or older, regular attendance at the health center (at least once at every 6 months), a diagnosis of hypertension 1 year prior to the completion of the study. The definition of hypertension was based on the JNC 6 guidelines (Joint National Committee on Prevention, Detection, Evaluation and Treatment of High Blood pressure 6).The samples were divided between men and women. This sample size is the minimum needed for the purpose of meeting the requirements in order to estimate the parameters under the maximum likelihood statistical procedures were used herein [18,19]. All the patients were asked to express their personal goals with respect to their hypertension. Only patients who were volunteers and their goal was to reduce the blood pressure, were included in the study. The Health centers were selected by cluster sampling in Yazd city and the patients were selected by a simple sampling method in the Health centers.

For data collection, we used a questionnaire that was previously used by Taylor et al [13] but we changed some questions of our questionnaire to be similar to the culture of our samples. The questionnaire was divided in 3 sections: 1–demographic questions 2–questions about pressure of samples and 3–questions about MGB. Attitude toward trying was measured with seven 5–point items. Subjective norms were measured with eight 5–point items. Anticipated emotions (positive emotion and negative emotion) were measured with fifteen 5–point items. Perceived Behavior Control (PBC) was measured with three 5–point items. Desires were measured with two 5–point items. Trying was measured with two 5–point items. Intention was measured with two 5–point items.  Finally, the past behavior was measured with five 5–point items. 

To ensure the clarity of the questionnaire, pilot testing of the questionnaire was also performed by using the coherence and consistency of 30 participants who were not included in the survey.

Afterwards, the questionnaire was modified based on their feedback. Content validity was established by 5 experts who were academic staff and health educators. The reliability and validity of the questionnaire was determined and its Alfa cronbach was (a=0.83).

All the data were transferred directly into SPSS (Statistical Package for Social Sciences). The level of confidence interval was of 95%

We obtained an informed consent from all the participants and, in addition, the managers of Health centers assured the participants that their responses were confidential.

Of 200 participants, 45.5% were men and 54.5% were women. The mean age was (59.8[+/–]11.9) 30–86 years old. A total of 8.5% had diploma or completed some university education, 9.5% studied 6–12 years of school (did not graduate with diploma), 28.5% studied in primary school (less than six years of school) and 53.5% were illiterate. The mean number of years with hypertension was (8.7[+/–]7.6). 86.5% reported adherence to their anti–hypertensive medications. With respect to stated goals, 61.5% of them indicated that their goal was to reduce their blood pressure and 35.5% had the goal to maintain their current blood pressure. The participants in this study were approximately 52% housewives, 10% government personnel, 19.5% free workers and 18.5% unemployed

## Results

Men and women showed moderately high means for attitudes, subjective norms, and positive anticipated emotions toward trying to reduce or maintain their blood pressure. Men showed moderate means for perceived behavioral control, past behavior, desire, trying and intention to reduce or maintain their blood pressure. Women showed lower moderate mean in perceived behavioral control, past behavior, desire, trying and intention to reduce or maintain their blood pressure. The mean of negative anticipated emotion was lower than the mean in men and women. There was a significant difference between all the constructs of MGB, gender and self–regulation of their hypertension, unless the negative anticipated emotion. (Table 1)

**Table 1:** Appearance rate on 100,000 persons/year of hypopharynx cancer regarding sex and rase (after Canto and Devesa 2002).

Sex	Male	Female	P–value
Constructs of MGB	Mean[+/–]SD	Mean[+/–]SD	
Attitude	20.86[+/–]3.49	18.51[+/–]4.02	0.001
Subjective norms	27.21[+/–]4.7	25.55[+/–]6.55	0.047
Positive anticipated emotion	14.54[+/–]3.21	16.32[+/–]4.7	0.031
Negative anticipated emotion	11.49[+/–]2.01	11.54[+/–]2.01	0.87
Perceived behavior control	8.63[+/–]2.6	7.06[+/–]2.45	0.001
Past behavior	13.56[+/–]5	9.18[+/–]5.96	0.001
Desire	7.58[+/–]1.62	6.82[+/–]2.08	0.005
Intention	7.28[+/–]1.79	5.57[+/–]2.18	0.001
Trying	15.27[+/–]4.32	12.6[+/–]4.35	0.001

With respect to education, the data showed there is a significant difference between the trying intention for self–regulation of blood pressure, and the level of education of participants with p<0.001, it is significant for a perceived behavior control which is (p<0.008) and for positive anticipated emotion which is (p<0.039).The mean grade scores for trying, of the subjects in guidance and high school education, in self–regulation for decreasing or maintaining their blood pressure, was more than (17.05[+/–]3.82) others and in illiterate participants it was lower than others (13.12[+/–]4.23).(Table 2). 

**Table 2:** Distribution of mean grades of constructs of MGB related to their education

Education	Illiterate(N=107)	Primary(N=57)	Guidance and high school(N=19)	Diploma and university(N=17)	p–value
Constructs of MGB	Mean[+/–]SD	Mean[+/–]SD	Mean[+/–]SD	Mean[+/–]SD	
Attitude	19.32[+/–]3.92	19.54[+/–]3.76	19.52[+/–]4.42	21.29[+/–]4.2	0.306
Subjective norms	26.22[+/–]6.48	25.64[+/–]4.83	26.63[+/–]5.28	28.64[+/–]4.97	0.318
Positive anticipated emotion	16.23[+/–]4.69	17.1[+/–]3.25	17.84[+/–]3.09	19[+/–]3.14	0.04
Negative anticipated emotion	11.49[+/–]2.27	11.73[+/–]1.26	11.42[+/–]1.38	11.05[+/–]3.82	0.659
Perceived behavior control	7.55[+/–]2.5	7.31[+/–]2.58	8.94[+/–]2.34	9.35[+/–]3.14	0.008
Past behavior	11.57[+/–]5.42	9.59[+/–]6.32	12.84[+/–]6.33	12.11[+/–]7.07	0.092
Desire	7.14[+/–]1.91	7.05[+/–]1.75	7.73[+/–]1.59	7.11[+/–]2.78	0.598
Intention	6.1[+/–]2.18	6.05[+/–]2.08	7.57[+/–]1.92	7.52[+/–]2.12	0.003
Trying	3.12[+/–]4.23	13.28[+/–]4.56	17.05[+/–]3.82	16.82[+/–]4.7	0.001

The data showed there is a significant difference between the trying of the government personnel and that of the housewives' with p<0.001, so the mean grade scores of trying for the self-regulation of their blood pressure in government personnel was (15.9[+/–]4.08) and in housewives was (12.49[+/–]4.33)

Significant effects on desire were found for attitude (p<0.01), for PAE (p<0.01), for subjective norms (p<0.01), for PBC (P<0.01), for past behavior (p<0.01) and for trying (p<0.01). There was no significant difference between desire and NAE. The data showed there was a significant difference between all the constructs of MGB and trying and the intention for self–regulation of blood pressure (P<0.01) unless NAE. (Table 3). Results showed that the explainable variance for trying was of 52%, intention 50% and desire 29%. (Table 4).

**Table 3:** Rate of correlation of trying for self– regulation in blood pressure and constructs of MGB (*p<0.01; **p<0.05; NS)

Constructs of MGB	Trying	Attitude	SN	PAE	NAE	PBC	Past behavior	Desire to self–regulation
Attitude	0.586*	–	–	–	–	–	–	–
Subjective norms	0.383*	0.353**	–	–	–	–	–	–
Positive anticipated emotion	0.520*	0.38*	0.330*	–	–	–	–	–
Negative anticipated emotion	0.001	–0.113	0.069	0.057	–	–	–	–
Perceived behavior control	0.658*	0.549*	0.302*	0.398*	0.060	–	–	–
Past behavior	0.475*	0.422*	0.16**	0.290*	0.005	0.505*	–	–
Desire to self–regulation	0.452*	0.424*	0.331*	0.372*	–0.031	0.423*	0.262*	–
Intention	0.0623*	0.623*	0.270*	0.447*	–0.098	0.597*	0.530*	0.511*

**Table 4:** Indexes of analyses regression of MGB

Independent variables	beta	p–value	R^2^	Dependent variables
Perceived behavior control	0.325	0.001		
Past behavior	0.092	0.134	0.521	Trying
Intention	0.325	0.001		
Perceived behavior control	0.325	0.001	0.500	
Past behavior	0.287	0.001		Intention
Desire	0.298	0.001		
Attitude	0.194	0.015		
Subjective norms	0.146	0.033		
Positive anticipated emotions	0.169	0.017	0.290	Desire
Negative anticipated emotions	0.263	0.793		
Perceived behavior control	0.202	0.013		
Past behavior	0.005	0.946		

## Discussion

In this study, we understood how decision–making processes occur in the self–regulation of blood pressure. In this study, the mean number of years with hypertension was (8.7[+/–]7.6). 86.5% reported adherence to their anti–hypertensive medications. With respect to the stated goals, 61.5% indicated that their goal was to reduce their blood pressure and 35.5% had the goal to maintain their current blood pressure. The results of Stephanie [13] revealed that the mean years with hypertension in their participants was (18[+/–]11.2), that being more than our results. Perhaps the years with hypertension in our participants are more than this, but they did not refer to clinic and did not know about their hypertension before. In that study, 48% indicated that their goal was to reduce their blood pressure, 44% to maintain their current blood pressure; the conclusion is that these results are nearly the same as our results. In that research, the participants who reported an adherence to the anti–hypertensive medications were 67%, fewer participants than in our study.  

We saw a significant difference between all the constructs of MGB and the gender of the participants, unless a negative anticipated emotion occurred. Men and women showed moderately high means for attitudes, subjective norms, and positive anticipated emotions toward trying to reduce or maintain their blood pressure. Perceived behavioral control, past behavior, desire, trying and intention to reduce or maintain blood pressure mean scores in women were lower than in men. The mean of negative anticipated emotion was lower in men and women. However, Stephanie et al [13] described that men and women showed moderately high means for attitude, subjective norms, desire, intentions, and positive anticipated emotions toward trying to reduce or maintain their blood pressure. Moderate means were found on negative anticipated emotions, perceived behavior control, past behavioral control, past behavior, and the attempt to reduce or maintain their blood pressure. None of the means differed significantly across gender or goal. The results of our study related to education are not consistent with the results of the study in Michigan [13], as, from the total participants of our study 8.5% had diplomas and completed some university education, 9.5% partly graduated from high school and guidance school, 28.5% graduated from a primary school and 53.5% were illiterate. In that study, a total of 43.5% were university graduates, 31.3% graduated some university, 21.5% were high school graduates and 3.8% did not graduate from high school. 

In a study, carried out by Jackson [20], it was revealed that in order to predict the behavior, the MGB must be corrected and the past behavior must be added to it. Our results showed that the past behavior is a good predictor of intention for a behavior that is consistent with the Jackson's study. 

Our results showed that the past behavior, attitude, subjective norms, perceived behavior control and desire to self–regulation have an influence on the intention of reducing or maintaining their blood pressure. These results are consistent with the results of Hagger et al [21], they revealed that past physical activity behavior would attenuate the influence of attitude, subjective norms, perceived behavior control and self–efficacy on intention. However, they are in contrast with the Normans' study [22] that revealed weaker relationships, which were observed along with the increasing frequency of the past behavior.

In the present study, negative anticipated emotions had not influence on the intention to control the blood pressure; these are the similar results to the ones of Chapman [23], who showed that the anticipated level of emotions differed systematically from the experienced emotions, so that the vaccinated individuals anticipated more regret and less worry than they actually experienced.   

Results of our study indicate that the MGB parameter estimates and explains variance. The explained variances in perceived behavior control (beta= 0.325, p<0.001), past behavior (beta= 0.092, p=0.134) and intention (beta= 0.325, p<0.001) in trying to decrease and maintain blood pressure was of 52%. The explained variances in perceived behavior control (beta= 0.325, p<0.001), past behavior (beta= 0.287, p=0.001) and desire (beta= 0.298, p<0.001) for the intention of decreasing and maintaining blood pressure was of 50% and explained variances in perceived behavior control (beta= 0.202, p<0.015), past behavior (beta= 0.005, p=0.946) NAE (beta=– 0.263, p=0.793), PAE (beta =0.169, P=0.017), subjective norms (beta = 0.146, P=0.033) and attitude (beta =0.194, p=0.015) for the desire of decreasing and maintaining blood pressure was of 29%.

In a study carried out by Bagozzi et al [17], the explainable variance in attitude (beta=0.43, p<0.08) and subjective norms (beta=0.18, p<0.07) for the desire in self–regulation of their weight was of 47%, that is better than the results of our study. Many results of our study in this section are consistent with the results of study that was carried out in Michigan [13].

We can indicate that the behavior of hypertension control, could improve about 52% of the blood pressure, but we must pay attention to the hidden aspect of this behavior and improve them. This way, if we manipulate the Attitude, Subjective Norm and Emotion of patients and they improve their Desire, we cannot only control the hypertension 30% more than before, but we can also make a better substructure to change the  patients' behavior  
